# STV‐Na attenuates lipopolysaccharide‐induced lung injury in mice via the TLR4/NF‐kB pathway

**DOI:** 10.1002/iid3.770

**Published:** 2023-01-18

**Authors:** Yanhong Xu, Xiaoming Liu, Zhihui Zhang

**Affiliations:** ^1^ Department of Respiratory The First Affiliated Hospital of Zhengzhou University Zhengzhou China; ^2^ Department of Plastic Surgery, The First Affiliated Hospital of Xinjiang Medical University Xinjiang Medical University Xinjiang Urumqi China

**Keywords:** ALI, inflammation, oxidative stress, STV‐Na

## Abstract

**Background:**

Acute lung injury (ALI) is a potentially fatal disorder that is largely caused by inflammation. Sodium isostevanol (STV‐Na) is a terpenoid produced from stevioside, which possesses anti‐inflammatory and antioxidative stress characteristics. nevertheless, it is still unclear how STV‐Na affects ALI. Therefore, we investigated the possible STV‐Na therapeutic impacts on lipopolysaccharide (LPS)‐induced (ALI).

**Methods:**

We employed hematoxylin‐eosin staining to observe the impact of STV‐Na on lung histopathological alterations and used kits to detect the oxidative stress status of lung tissues, such as superoxide dismutase, malondialdehyde, and glutathione. The reactive oxygen species and myeloperoxidase expression in the tissues of lung was assessed by immunofluorescence and immunohistochemistry. Additionally, we detected the impact of STV‐Na on inflammatory cell infiltration in lung tissue using Wright‐Giemsa staining solution and immunohistochemistry, which was found to reduce inflammation in lung tissue by enzyme‐linked immunosorbent assay. Finally, using WB, we examined the impact of STV‐Na on the TLR4/NF‐kB pathway.

**Results:**

We observed that STV‐Na attenuated lung histopathological alterations in LPS‐induced lung damage in mice, reduced infiltration of inflammatory cell and oxidative stress in the tissue of lung, and via the TLR4/NF‐kB pathway, there is a reduction in the inflammatory responses in mouse lung tissue.

**Conclusions:**

These outcomes indicate that the response of inflammatory cells to LPS‐induced ALI in mice was attenuated by STV‐Na.

## Graphical abstract

Sodium isostevanol attenuates LPS‐induced lung inflammation in mice via the TLR4/NF‐κB signaling pathway.

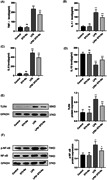

## 1 INTRODUCTION

Acute respiratory distress syndrome (ARDS) and acute lung injury (ALI) are the primary causes of death in intensive care unit individuals due to their persistently high mortality rates.^1^ There are various causes of ALI, and the most common cause is pneumonia caused by various pathogens, especially in the last 2 years. The new coronavirus caused acute lung injury that not only caused physical damage and sequelae to patients, but also caused an economic burden to society.^2^, ^3^, ^4^ Several drug studies have been conducted in clinical studies for ARDS, such as glucocorticoids, anti‐inflammatory medications and antioxidants; however, there are presently no effective drug therapies to treat these diseases.^5^ The current treatment for ALI is mainly based on supportive therapy, such as ventilation and fluid replacement, so it is particularly important to find new drug candidates for ALI treatment.^6^, ^7^

Diffusion of alveolar injury and disruption of the epithelial‐endothelial barrier is the leading cause of ALI, which leads to excessive infiltration in the interstitium and alveoli due to accumulation of protein‐rich fluid and blood cells. Tissue injury induce neutrophils migration, which are activated and, engaged with alveolar macrophages, platelets and other inflammatory and fixed lung cells, producing a variety of substances that aggravate inflammation.^8^, ^9^, ^10^ Oxidative stress due to inflammation also exacerbates lung injury even further, and there is significant indications that elevated reactive oxygen species (ROS) production in lung endothelial cells leads to lung barrier integrity reduction, induced by factors such as tumor necrosis factor or LPS.^11^, ^12^

Toll‐like receptors are a class of 10 closely similar transmembrane receptors, of which TLRs 1, 2, 4, 5 and 6 are situated on the cell membrane, while TLRs 3, 7, 8, and 9 are situated inside the cell.^13^ Lipopolysaccharides, a Gram‐negative bacterial cell wall portion, contain lipid A, an oligosaccharide “core” that is not repeating, and distal polysaccharides. TLR4 identifies the endotoxic characteristics of lipid A, which is a typical PAMP, making TLR4 particularly important.^14^ Studies have demonstrated that the binding of endotoxin to extracellular binding proteins facilitates the binding of endotoxin to TLR4 thereby regulating the cytokines expression with proinflammatory properties by NF‐κB pathway activation.^15^ NF‐κB/P65 mechanism activation controls the release of proinflammatory cytokines.

Sodium isosteinol (STV‐Na) is a terpenoid extracted from stevioside which have antioxidant, antitumor and anti‐inflammatory properties, as well as a reliable safety profile, and it has shown good protective effects in colitis and myocardial studies. According to previous studies. it was found that STV‐Na inhibited macrophage polarization to reduce inflammatory cytokine production and attenuated LPS‐induced multiorgan damage. Despite this, there are not many studies on the potentiality of STV‐Na therapeutic effects for ALI.^16^, ^17^, ^18^ Prior research have confirmed that STV‐Na can reduce the activity of NF‐ κB/p65.^18^ We therefore utilized a LPS‐induced acute lung injury model and explored whether STV‐Na has a potential therapeutic impact on lung injury and the intrinsic mechanisms associated with it. These documents demonstrate a potential basis for STV‐Na treatment of ALI.

## 2 MATERIALS AND METHODS

### 2.1 Reagents and chemicals

STV‐Na was provided by Key‐Pharma Biological Inc. LPS (*Escherichia coli* 0111: B4) was retrieved from Sigma Chemical Co. myeloperoxidase (MPO) antibody, F480 antibody, GAPDH antibody, NF‐κB antibody, p‐NF‐κB antibody and 4,6‐diamino‐2‐phenyl indole (DAPI) were obtained from Abcam. TLR4 antibody was obtained from Cell Signaling Technology. Inc. ROS fluorescent probes were purchased from Bestbio Biologicals Ltd. The anti‐rabbit immunoglobin G (IgG) antibody was purchased from Zhongshan Jinqiao Biological Co. ExCell Bio Co was the supplier of all enzyme‐linked immunosorbent assay (ELISA) kits. Wright–Giemsa staining solution, hematoxylin‐eosin (HE) Staining Kit, bicinchoninic acid (BCA) Protein Assay Kit, 5% bull serum albumin (BSA) Blocking Buffer, goat serum and Tris buffered saline containing 0.1%Tween 20 (TBST) were acquired from Solaibao Life Sciences Co. (Beijing, China).

### 2.2 Animal model and treatment

Male BALB/c (20–25 g, eight weeks) mice were obtained from Beijing SPF Biotechnology Co. Following the Guide for the Care and Use of Laboratory Animals, all experiments were undertaken (NIH Publication No. 85‐23, revised 1996) and was accepted by the PLA General Hospital Animal Ethics Committee. Mice were placed under standard circumstances (20 ± 2°C, 50%–65% humidity, 12:12 h dark/light cycle) given free aceess to water and food, as well as adaptation to fed for 1 week before the study was conducted. Randomly, mice were separated into 4 groups of 6 mice each and grouped as follows: (1) saline + saline, control; (2) saline + STV‐Na, STV‐Na; (3) LPS + saline, LPS; and (4) LPS + STV‐Na. Mice were injected intraperitoneally with LPS (20 mg/kg) or saline (0.1 ml/10 g). After 12 h, mice were injected intraperitoneally with saline (0.1 ml/10 g) or STV‐Na (20 mg/kg) every 12 h for 2 days, and on the third day, lung function tests and samples were taken 3 h following the initial intraperitoneal dose.

### 2.3 Lung function test

The mice were anesthetized with pentobarbital sodium (90 mg/kg) to suppress their spontaneous breathing, and then the trachea was exposed. The pulmonary function test apparatus was attached to the trachea of the mice based on a frequency set to 90, respiratory quotient set to 15:10, and tidal volume set to ml/kg. Then, lung function‐related parameters, such as airway resistance (RAW), pulmonary dynamic compliance (Cdyn) and peak expiratory flow (PEF), were recorded.^19^

### 2.4 Alveolar lavage fluid measurement

A 1 ml of ice‐cold PBS was used to obtain the alveolar lavage fluid, the lungs were repeatedly flushed, and under conditions of centrifugation at 200*g* for 10 min at 4°C, the fluid was obtained. The alveolar lavage fluid protein concentration was estimated utilizing BCA reagents. Utilizing a hemocytometer, researchers were able to determine the absolute number of cells with nuclei in the alveolar lavage fluid. Cell pellets were immersed in 150 μl PBS, and Wright–Giemsa staining solution was used to stain the cell suspension that had been applied on coverslips. Cells were examined with a light microscope (Olympus). Leukocytes, neutrophils and macrophages were then counted.

### 2.5 ELISA

A working solution contain Biotinylated antibody was supplemented to the sample wells and standard wells and incubated for 120 min at room temperature. Following five washes, each well received 100 μl of working solution conjugated enzyme and incubated for 60 min at room temperature. The plate was rinsed five more times, each well received 100 μl of chromogenic substrate, which was incubated for 15 min at room temperature. Eventually, 100 μl of termination solution was supplemented to the reaction wells, and at 450 nm, the absorbance value was measured. Using the standard curve, the sample's concentration was calculated.

### 2.6 Hematological analysis

Peripheral blood from each group of mice was collected in ethylene diamine tetraacetie acid‐containing tubes and then examined for the count of white blood cell (WBC), lymphocyte (LYMPH), monocyte (MONO), neutrophil (NEUT), and red blood cell (RBC), as well as (HCT) hematocrit using a hematological analyzer (IDEXX).^18^

### 2.7 Malondialdehyde (MDA), superoxide dismutase (SOD), catalase (CAT), and Glutathione (GSH) determination

Lung tissues were rinsed with ice‐cold PBS, lysed in lytic solution, and centrifuged for 10 min at 8000*g*, and assayed according to the requirements of the manufacturers of SOD, MDA, GSH, and CAT reagents. Finally, the absorbance of MDA at 532 and 600 nm, GSH at 412 nm, SOD at 560 nm and CAT at 240 nm were measured.^20^

### 2.8 ROS detection

The prepared tissue sections were three times washed with ice‐cold PBS, diluted with a DHE reactive oxygen probe to 10 μM, evenly added dropwise onto the tissue sections, incubated for 30 min in a 37°C incubator, washed three times again with PBS, sealed with a DAPI‐containing sealing reagent and observed under a fluorescence microscope.^21^

### 2.9 Wet/dry (W/D) ratio

Pulmonary edema degree is indicated by the W/D ratio. The right lung of mice was weighed after euthanasia and then reevaluated after 72 h of dryness in a 60°C oven.^20^

### 2.10 Lung histopathology analysis

Tissues of lung were fixed with 4% paraformaldehyde for 2 days, then immersed using paraffin, split into 5‐μm sections, adhered to slides, smeared with hematoxylin and eosin (HE) and investigated under a microscope. HE sections of lung tissue were scored and recorded in accordance with the lung injury scoring criteria without the knowledge of the pathologist of the experimental group. Therefore, the criteria were set based on injury (%): 0 points for 0% injury, 1 for 25%, 2 for 50%, 3 for 75%, and 4 for 100% injury.^22^

### 2.11 Immunohistochemical staining

Using a microtome, paraffin‐embedded lung tissue was sliced into 5‐μm slices, and the slices were treated with antigen repair and incubated with 3% of H_2_O_2_ for 10 min at room temperature to suppress endogenous peroxidase. Slices were rinsed with PBS and blocked with goat serum for 1 h. Primary antibody was added dropwise and incubated through the night at 4°C. Utilizing PBS, Sections were washed and then preserved with polymeric adjuvant and anti‐rabbit IgG for 20 min. The dishes were incubated at 37°C for 30 min. Slices were stained with DAB and examined by light microscope. When the cytoplasm turned brown, the process was stopped by adding tap water. Tissues were retreated with hematoxylin, dehydrated, clarified, stamped with gum, and then examined under a microscope.

### 2.12 Western blot analysis

Using lysis solution, tissues of lung were collected and lysed. On 8%–12% SDS polyacrylamide gels, lung tissue lysate proteins were separated by electrophoresis and electronically placed to a membrane of nitrocellulose, closed with 5% BSA for 1 h, preserved through the night in a refrigerator at 4°C with primary antibody, rinsed three times with TBST for 5 min each, incubated with secondary antibody for 1 h and rinsed 3 times with TBST over and over. The signals were visualized with an improved chemiluminescence detection system. Densitometric analysis was conducted with Image J.

### 2.13 Statistical analysis

The outcomes are displayed as the mean ± standard deviation (SD). There were six animals per group, and statistical analysis was conducted utilizing Prism 8.02 software (GraphPad software). Using an independent two‐tailed Student's *t* assay, the two groups were compared. If a comparison were conducted to more columns, a one‐way analysis of variance was performed, with *p* < .05 representing statistical significance.

## 3 RESULTS

### 3.1 STV‐Na attenuates the pathological changes of LPS‐induced ALI

**Figure 1:**
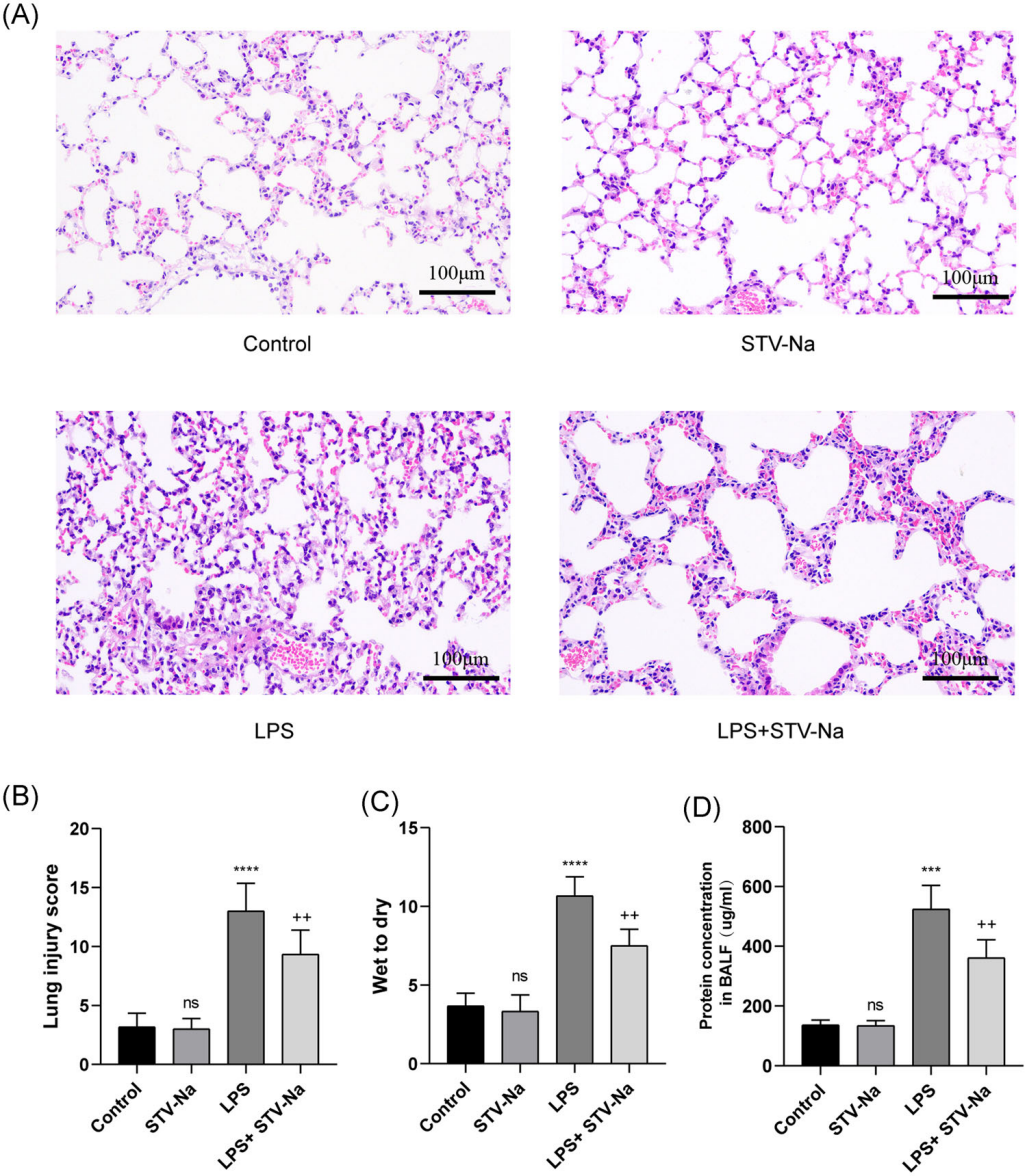
STV‐Na attenuates the pathological changes of LPS‐induced acute lung injury. (A) Representative photographs of H&E staining of lung tissues in each group. Scale bars = 100 µm. (B) The lung injury score and (C) the ratio of wet to dry were assessed. (D) The protein concentration in BALF was measured by a BCA assay. The data are shown as the mean ± SD (*n* = 6), ns, no significance versus control; ****p < .0001; ***p < .001 versus control; ^++^ *p* < .01 versus LPS.

**Figure 2:**
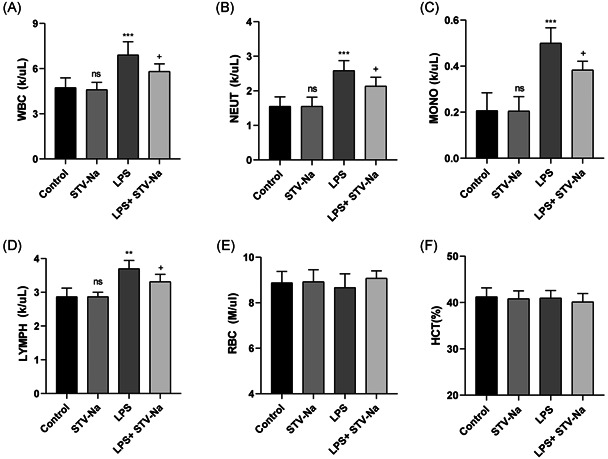
STV‐Na restores hematological and biochemical parameters in mice with acute lung injury. The numbers of (A) white blood cells, (B) neutrophil, (C) monocyte, (D) lymphocyte, (E) red blood cells and (F) hematocrit values were detected. The data are shown as the mean ± standard deviation (*n* = 6), ns, no significance versus control; ***p < .001; **p < .01 versus control; ^+^ *p* < .05 versus LPS.

LPS was utilized to produce ALI in mice to study the impact of STV‐Na on ALI. The pathological state of lung tissue of mice in every group was analyzed by HE staining. According to Figure 2A,B, normal mice were cured with STV‐Na, and there were no obvious side effects. The structure of alveoli in mice in the LPS‐induced model group was disordered. After therapy with STV‐Na, lung injury was significantly alleviated, and interstitial edema was improved. Figure 1C shows the ratio of lung W/D weight in each group. In comparison with the control group, the proportion of mice cured with LPS was significantly elevated, but it was greatly reduced by STV‐Na treatment. In addition, we discovered that STV‐Na therapy significantly decreased the concentration of proteins in BALF. (Figure 1D).

### 3.2 STV‐Na restores hematological and biochemical metrics in mice with ALI

The systemic inflammation degree is frequently showed in an abnormal hematologic metrics.^23^ Hematological parameter analyses in this research indicated that leukocytosis was observed in the LPS group (Figure 2A), over production of granulocytes (Figure 2B), monocytosis (Figure 2C), lymphocytosis (Figure 2D), and they were all remarkably ameliorated by STV‐Na treatment. However, the RBC (Figure 2E), and HCT (Figure 2F) parameters of mice were not significantly changed. STV‐Na appears to show suppression impacts on systemic inflammation and anemia according to the hematological metrics outlined above in ALI mice.

### 3.3 Lung function of ALI mice was enhanced by STV‐Na

**Figure 3:**
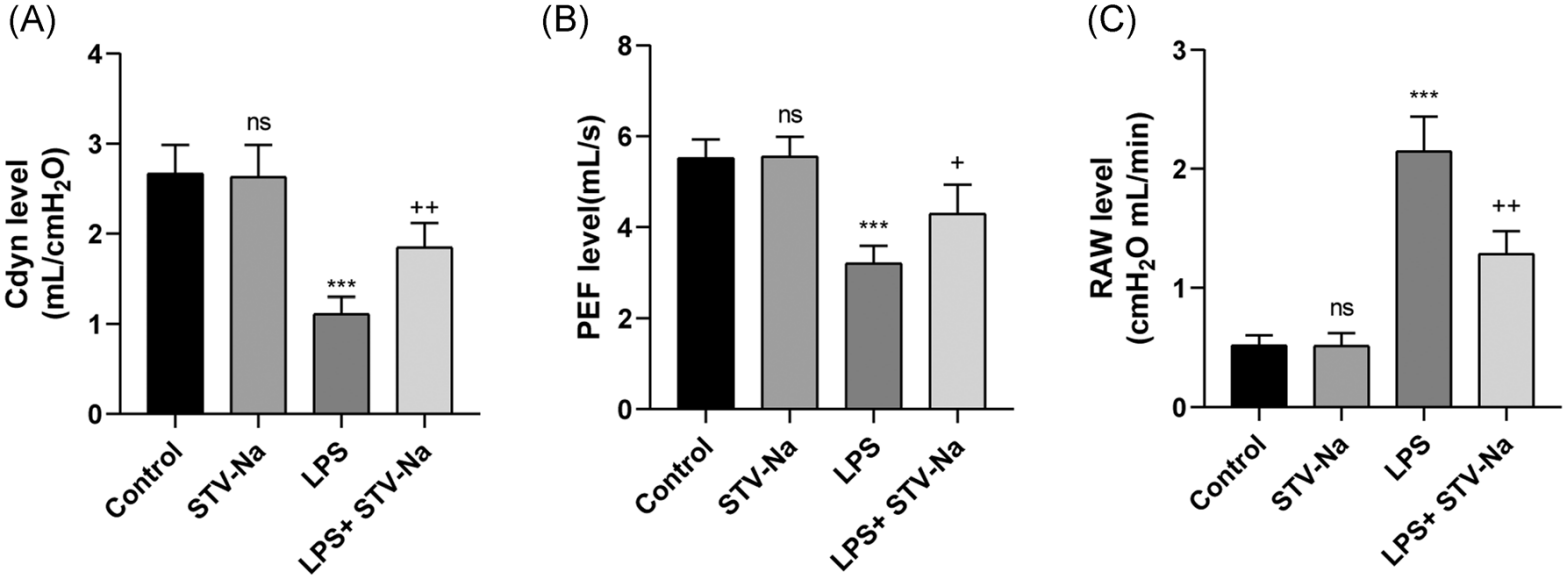
Effects of STV‐Na on lung function in LPS‐induced acute lung injury mice. (A) Cdyn (ml/cmH_2_O), (B) PEF (ml/S), and (C) RAW (cmH_2_O ml/min) were measured in LPS or saline‐treated mice after 72 h. The data are shown as the mean ± SD (*n* = 6), ns, no significance versus control; ***p < .001 versus control; ^++^ *p* < .01; ^+^ *p* < .05 versus LPS.

We detected the pulmonary dynamic compliance (Cdyn) of mice in each group and found that the Cdyn of mice in the model of LPS group reduced significantly, and STV‐Na treatment alleviated this symptom (Figure 3A). We also found that the decrease in PEF induced by LPS stimulation could be restored after STV‐Na treatment (Figure 3B). According to Figure 3C, in comparison with the control group, airway resistance (RAW) in the LPS model group was significantly increased, which could be effectively relieved by STV‐Na therapy. These findings demonstrated that injection of STV‐Na significantly improved lung function that had been compromised by LPS.

### 3.4 STV‐Na reduces LPS‐induced oxidative stress in mouse lung tissue

**Figure 4:**
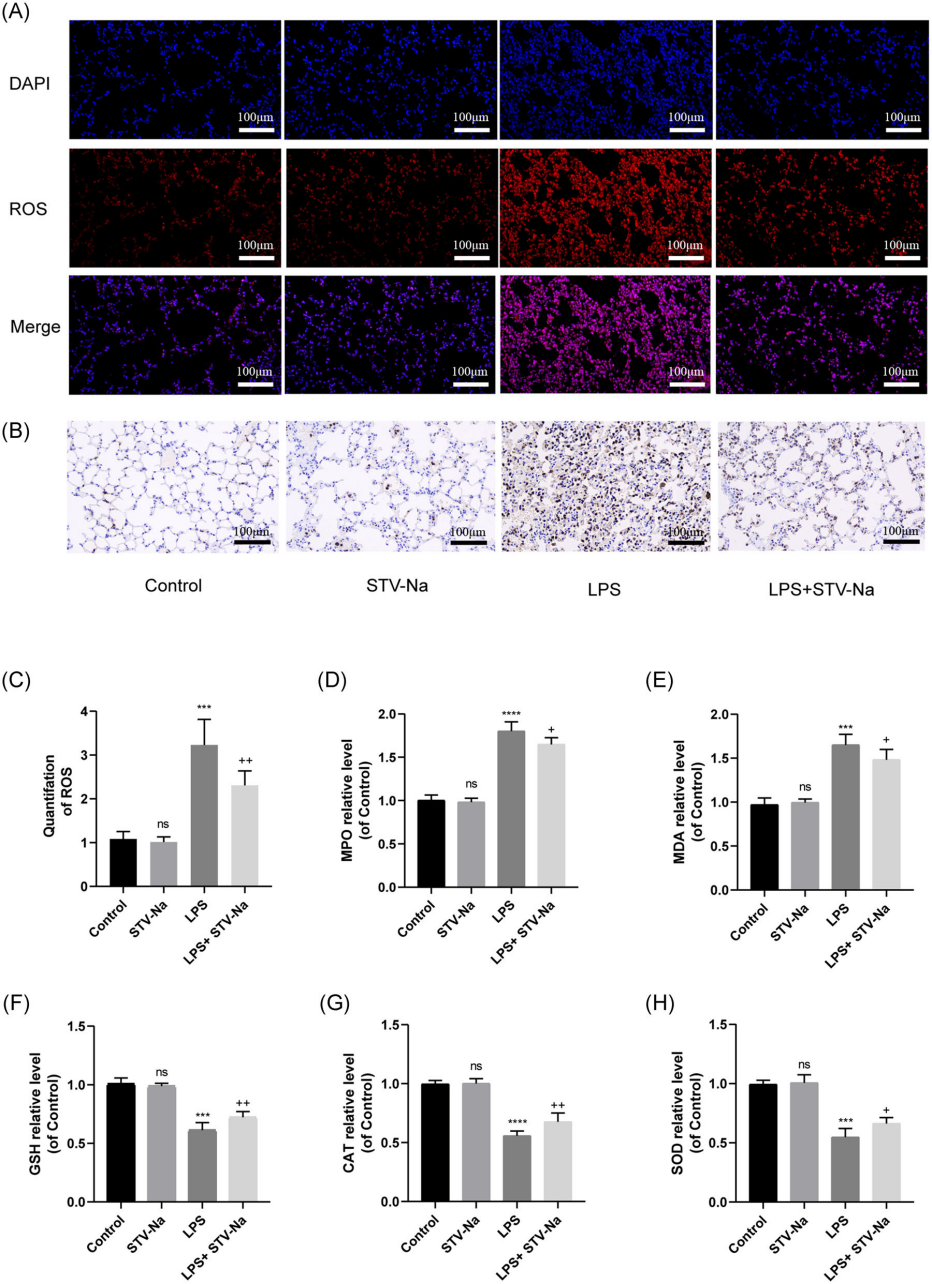
STV‐Na reduces LPS‐induced oxidative stress in mouse lung tissue. (A) Detection of lung tissue ROS levels by a DHE probe. The scale bars are 100 µm. (B) Lung tissue MPO levels were detected by immunohistochemistry. Scale bars  = 100 µm. (C) Quantitative histogram of ROS. (D–H) MPO, malondialdehyde, Glutathione, catalase, and superoxide dismutase were detected by enzyme‐linked immunosorbent assay. The data are shown as the mean ± standard deviation (*n* = 6), ns, no significance versus control; ****p < .0001; ***p < .001 versus control; ^++^ *p* < .01; ^+^ *p* < .05 versus LPS.

Given that reactive oxygen species are one of the distinguishing hallmarks of ALI, we used immunofluorescence staining to investigate the potential impact of STV‐Na on oxidative stress caused by LPS in mice. As illustrated in Figure 4A,C, LPS induction increase the development of ROS in mouse lung tissue, and STV‐Na significantly reversed this phenomenon. Additionally, the activity of MPO is believed to be a particular indication of NEUT infiltration and latent cases of ALI, so we used immunohistochemical staining to detect the level of MPO in mouse lung tissue. As shown in Figure 4B,D, the LPS‐induced mice had an obvious improve the activity of MPO, despite this activity was decreased by treatment with STV‐Na. MDA activity is a frequent oxidative stress marker.^24^ GSH, CAT, and SOD are predominantly enzymes have antioxidant effect that control oxidative stress in the lungs.^25^ From the results shown in Figure 4E–H, LPS‐induced mice exhibited a significant increase in MDA levels, which were significantly reduced by STV‐Na therapy. In addition, LPS‐induced mice exhibited substantial declines in the antioxidant enzyme activity (GSH, CAT, and SOD), which were greatly increased by STV‐Na therapy.

### 3.5 STV‐Na decrease the infiltration of inflamed cells into the tissues of lung

**Figure 5:**
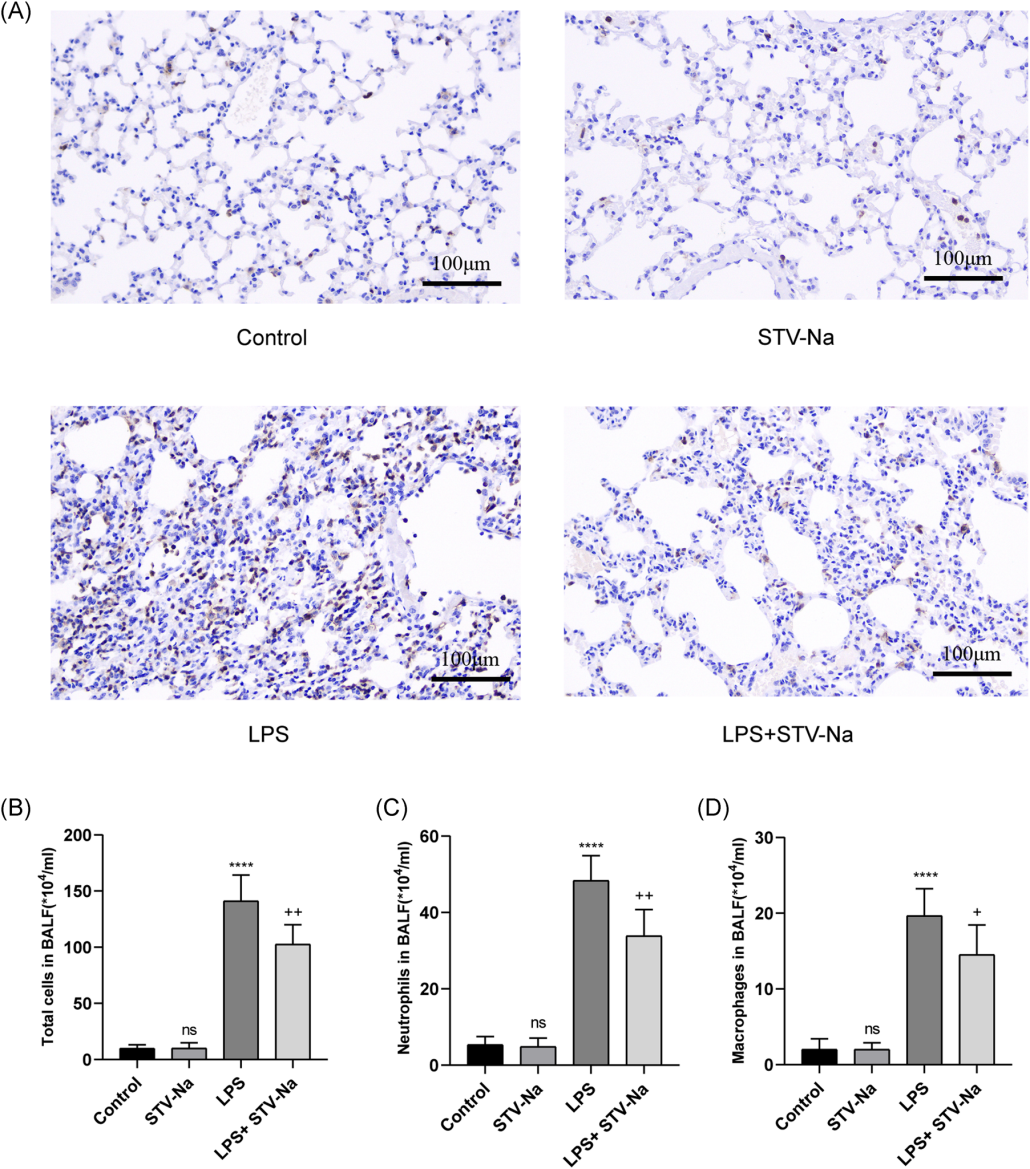
STV‐Na reduces the infiltration of inflammatory cells into lung tissue. (A) Expression of F4/80 in lung tissue using immunohistochemical detection. The scale bars are 100 µm. (B) Total cells, (C) neutrophils and (D) macrophages in BALF were assessed by Wright–Giemsa composite dyeing stain counting. The data are shown as the mean ± standard deviation (*n* = 6), ns, no significance versus control; ****p < .0001 versus control; ^++^ *p* < .01; ^+^ *p* < .05 versus LPS.

We performed immunohistochemical staining on lung tissue sections to detect the infiltration of macrophages (F4/80). As expected, the induction of LPS cause an elevation in the number of macrophages in lung sections, but this increase in the number of cells was reduced by STV‐Na treatment (Figure 5A). As shown in Figure 5B–D, THE total cell number, NEUT number and macrophage number in BALF were obviously elevated in the LPS group and were downregulated by STV‐Na therapy. These findings demonstrate the anti‐inflammatory effect of STV‐Na therapy.

### 3.6 STV‐Na suppresses LPS‐induced lung inflammation in mice by the TLR4/NF‐κB signaling pathway

**Figure 6:**
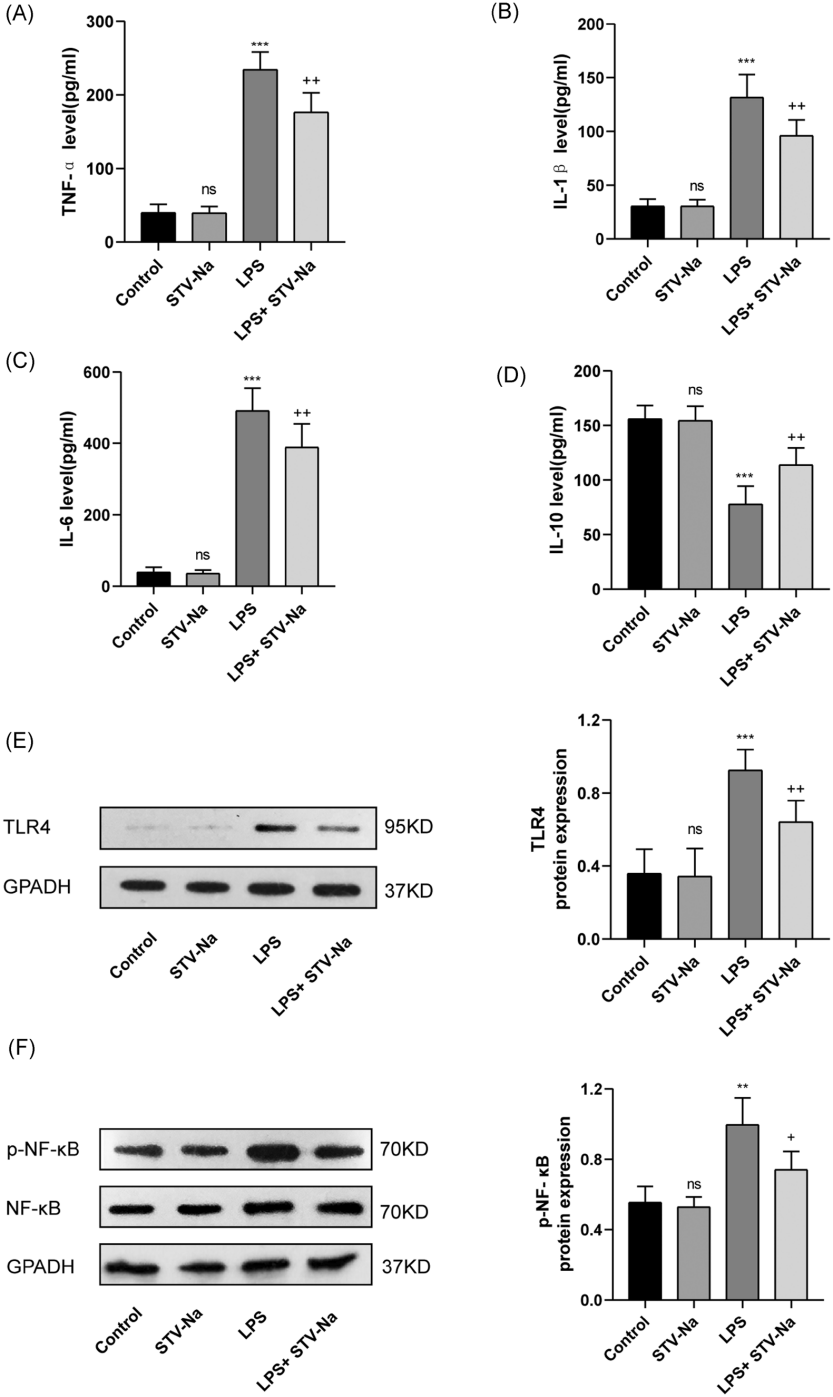
STV‐Na attenuates LPS‐induced lung inflammation in mice via the TLR4/NF‐κB signaling pathway. (A)TNF‐α, (B) interleukin (IL)‐1β, (C) IL‐6, and (D) IL‐10 were identified by enzyme‐linked immunosorbent assay. (E) TLR4 and (F) p‐NF‐κB were measured by western blot. The data are shown as the mean ± standard deviation (*n* = 6), ns, no significance versus control; ***p < .001; **p < .01 versus control; ^++^ *p* < .01; ^+^ *p* < .05 versus LPS.

Furthermore, study the anti‐inflammatory impact of STV‐Na, the proinflammatory cytokines levels in the BALF of mice in every group were recognized by ELISA. Endotoxin was found to considerably stimulate the release of TNF‐, interleukin (IL)‐6, and IL‐1, according to the previous findings. However, STV‐Na treatment could inhibit the expression of proinflammatory factors, which indicated that STV‐Na had significant anti‐inflammatory activity (Figure 6A–C). IL‐10 is an anti‐inflammatory factor, and we found that STV‐Na treatment increased its expression level (Figure 6D). We used WB to detect the condition of the TLR4/NF‐κB signaling pathway in every group of lung tissue. The values of TLR4 and p‐NF‐B p65 expression were found to be significantly increased under LPS activation but were highly inhibited by STV‐Na treatment (Figure 6E,F).

## 4 DISCUSSION

In this research, we explored the impact of STV‐Na on ALI. Although STV‐Na has been demonstrated to be healthy in the majority of clinical trials, there is little data demonstrating that long‐term use may cause adverse reactions. Previously, 10 and 20 mg/kg of STV‐Na were utilized to cure colitis or diabetic disorder for up to 9 weeks.^18^, ^26^ Considering our planned short‐term use, we embraced the 20 mg/kg dose scheme.

ALI is defined as damage to pulmonary capillary endothelial cells and alveolar epithelial cells caused by pathological disorders including infection, shock, trauma and scalding, creating diffuse pulmonary fibrosis and edema of alveoli, resulting in acute hypoxic respiratory failure.^27^, ^28^ A great deal of evidence shows that the infiltration of protein into the BALF and the lung W/D ratio indicate the alveolar/capillary barrier's integrity and the degree of pulmonary edema.^29^, ^30^ In our research, activation with LPS substantially developed the W/D ratio in mice lung tissue and triggered the release of protein into BALF, while STV‐Na efficiently alleviated this symptom. It was previously reported that white blood cells, such as macrophages and NEUT, in BALF increased significantly during LPS‐induced ALI and in patients.^31^, ^32^ Similarly, MPO activity was correlated with the quantity of NEUT entering the lung.^33^ Our findings demonstrate that STV‐Na therapy not only reduces the discharge of total cells, NEUT, and macrophages in BALF, but also inhibits the development in MPO levels in lung tissue stimulated by LPS. In addition, the lung tissue of mice stimulated by LPS can cause excessive ROS production and the formation of MDA, which can aggravate lung injury, and these oxidative products can be enhanced by the antioxidant enzymes GSH and SOD.^34^, ^35^, ^36^ These results showed that STV‐Na evidently developed the SOD activity and GSH, suppressing the ROS formation and MDA, which were stimulated by LPS. Therefore, we believe that STV‐Na can reduce LPS‐induced lung damage by inhibiting inflammation and oxidative‐related injuries. It should be noted that the current investigation lacks a positive anti‐ALI drug to examine the potency of STV‐Na. Anti‐inflammatory glucocorticoids researched extensively because of their effects on improving ALI.^37^ Numerous drugs, including captopril, rosiglitazone and incyclinide, have revealed good impact of treatment in various models of animal suffering from ALI.^38^ These medications will be utilized in our future studies. A strong inflammatory response is indicated by an increase in WBCs, NEUTs, LYMPHs, and MONOs.^39^ In this investigation, a substantial rise in WBCs was detected in blood samples of acute lung injury. As opposed to, WBCs, NEUTs, LYMPHs, and MONOs were reduced after supplementation with STV‐Na. These result further prove that STV‐Na has a satisfactory anti‐inflammatory effect.

NF‐κB signaling pathway contributing in regulation of LPS‐induced ALI inflammation.^40^, ^41^ We found that STV‐Na significantly suppressed the signaling pathway of TLR4/NF‐κB, thus blocking LPS‐induced NF‐κB phosphorylation and reducing the inflammatory response. The pathophysiological pathway of ALI is complex, and involves multiple signaling pathways. In our prospect work, we will further study the detailed molecular mechanism by which STV‐Na regulates the TLR4/NF‐κB signaling pathway. To gain a deeper comprehension of the STV‐Na therapeutic effect on ALI.

In this investigation, we utilized the LPS‐induced acute lung damage model to confirm the impact of STV‐Na treatment. This research also has the following limitations: Additionally, we must confirm the therapeutic efficacy in the treatment of STV‐Na on other lung injury models. What kind of immune cells is regulated by STV‐Na to alleviate acute lung injury needs to be further explored. And the long‐term use of STV‐Na, the toxic and side effects on organisms are also worthy of further study. In summary, the outcomes of this investigation suggest that the defensive mechanism of STV‐Na on LPS‐induced ALI may be because of its anti‐inflammatory and antioxidant properties, and its mechanism may be achieved by suppression of the NF‐κB signaling pathway. Consequently, STV‐Na can be employed as a candidate strategy for ALI treatment.

## AUTHOR CONTRIBUTIONS

Yanhong Xu conducted the laboratory experiments and drafted the manuscript. Xiaoming Liu was in charge of analyzing and interpreting the data. Zhang Zhihui was responsible for the diagram of the study and the revision of the calligraphy. The final calligraphy was read and accepted by all authors.

## CONFLICT OF INTEREST

The authors declare no conflict of interest.

## ETHICS STATEMENT

Every experiment was conducted under the supervision of the Care and Use of Laboratory Animals (NIH Publication No. 85‐23, revised 1996, USA) and was accepted by the PLA General Hospital Animal Ethics Committee.

## Data Availability

The data supporting the findings of this investigation are accessible upon reasonable request from the corresponding author.
